# Determinants of Vitamin D Status in Fair-Skinned Women of Childbearing Age at Northern Latitudes

**DOI:** 10.1371/journal.pone.0060864

**Published:** 2013-04-09

**Authors:** Linnea Hedlund, Petra Brembeck, Hanna Olausson

**Affiliations:** Department of Internal Medicine and Clinical Nutrition, Sahlgrenska Academy, University of Gothenburg, Gothenburg, Sweden; Baylor College of Medicine, United States of America

## Abstract

**Background and Objective:**

Poor vitamin D status during pregnancy has been associated with unfavorable outcomes for mother and child. Thus, adequate vitamin D status in women of childbearing age may be important. The aim of this study is to investigate the determinants of 25-hydroxyvitamin D (25(OH)D) serum concentrations in women of childbearing age living in Sweden, at latitude 57–58° north.

**Method:**

Eighty four non-pregnant, non-lactating, healthy, fair-skinned women aged between 25–40 years were included. All subjects provided blood samples, four day food records and answered questionnaires about sun exposure and lifestyle. Total serum 25(OH)D was analyzed using Roche Cobas® electrochemoluminiescent immunoassay.

**Results:**

Mean 25(OH)D was 65.8±19.9 nmol/l and 23% of the subjects had concentrations <50 nmol/l. Only 1% had concentrations <25 nmol/l. Determinants of 25(OH)D concentrations were recent sunbed use, recent travel to southern latitude, season, estrogen contraceptive use and use of supplementary vitamin D (R^2^ = 0.27).

**Conclusion:**

Every fifth woman had 25(OH)D concentrations <50 nmol/l. About 30% of the variation in vitamin D status was explained by sun exposure, use of vitamin D supplements and use of estrogen contraceptives. Cutaneous vitamin D synthesis seems to be a major contributor to vitamin D status, even at northern latitudes. Thus, recommendations on safe UV-B exposure could be beneficial for vitamin D status.

## Introduction

The association between vitamin D status and bone health is well established. Since the discovery of vitamin D receptors throughout the human body, interest in the non-skeletal benefits of vitamin D has increased [1]. For example, poor maternal vitamin D status during pregnancy has been associated with unfavorable outcomes for the mother such as delivery by cesarean section [2], preeclampsia [3] and gestational diabetes [4], as well as for the child. Outcomes for the child include low bone mineral content [5] and being small for gestational age [6]. Thus, adequate vitamin D status in women of childbearing age may be important.

There is no universally accepted definition of vitamin D deficiency, insufficiency or optimal status. Generally, serum 25-hydroxyvitamin D (25(OH)D) concentrations >50 nmol/l are considered desirable for fracture prevention [7] while levels <25 nmol/l are associated with rickets [8]. Some argue that optimal vitamin D status should be defined at higher 25(OH)D concentrations than 50 nmol/l [9].

The main dietary sources of vitamin D include meat, dairy products and fatty fish. Vitamin D can also be derived from supplements or be synthesized in the skin by exposure to ultra-violet (UV) light, primarily UV-B. However, there is insufficient UV-B radiation for cutaneous vitamin D production during winter north of latitude 35° north [10]. Use of sun protection factor can further inhibit cutaneous vitamin D synthesis as it may block part of the UV-B spectrum [10] while sunbeds can increase 25(OH)D concentrations by emitting UV-B radiation [11].

Previous studies on women living at northern latitudes have focused on populations at risk of hypovitaminosis D; pregnant [12], [13] postmenopausal women [14], [15] or women with high skin pigmentation and/or concealing clothing [16], [17]. Very few data are available on vitamin D status of non-pregnant, fair-skinned women of childbearing age living at northern latitudes [18], [19], [20], [21]. Only one study has previously investigated determinants of vitamin D status in Swedish women of childbearing age and that study is rather old [18]. The knowledge that lower vitamin D status has been associated with unfavorable pregnancy outcomes emphasizes the need to investigate the vitamin D status and its determinants in women of childbearing age, especially at northern latitudes where cutaneous vitamin D synthesis is limited. Understanding the determinants of vitamin D status is necessary to increase the possibilities of improving vitamin D status when needed. This warrants an examination of the impact of the individual factors influencing vitamin D status, particularly the modifiable factors relating to lifestyle and behavior. The purpose of the present study is to examine vitamin D status in a group of fair-skinned Swedish women of childbearing age living at latitude 57–58° north and to determine the impact of dietary vitamin D intake, vitamin D supplement use, body mass index (BMI), contraceptive use and sun exposure.

## Materials and Methods

### Ethics Statement

This study was conducted according to the guidelines laid down in the Declaration of Helsinki and all procedures involving human subjects were approved by the Regional Ethics Committee in Gothenburg. Written informed consent was obtained from all subjects.

### Subjects

Subjects were originally recruited to participate in a longitudinal study investigating changes in bone metabolism and body composition during and after lactation. When enrolled, subjects were 25–40 years of age, and recruited either as pregnant subjects or as non-pregnant and non-lactating controls. Recruitment methods included advertisements on a webpage addressing pregnant women in western Sweden and posters in the vicinity of Gothenburg at maternity clinics and in public places, e.g. pharmacies. Inclusion criteria for the current cross-sectional study were that subjects had not been pregnant or lactating in the past six months. Of the 129 subjects recruited to the longitudinal study, 84 were eligible for the current study. Non-eligible subjects were excluded due to recent or current lactation, drop-out or missing food records.

### Study Protocol

All study visits were conducted in the morning while subjects were fasting. Venous blood samples were drawn and body weight (in underwear, Tanita, BWB-800MA, Rex Frederiksbergs Vaegtfabrik) and height (standardized wall stadiometer) were measured.

All subjects provided information on consumption of prescription medication, contraceptive use, parity and lactation. When applicable, smoking was defined as ≥1 cigarette per week.

Sun exposure was assessed using a questionnaire complied by Burgaz et al [14]. Subjects were asked to define their sun preferences (always in the sun, both in sun and shadow, never in the sun), sun protection factor usage (always, sometimes, never), skin type by using the Fitzpatrick’s scale I-IV [22] and sunbed use. Sunbed use was defined as recent if it had occurred in the last 30 days. Subjects were also asked if they had travelled to southern latitudes in the previous six months. If so, they were asked the duration and location of the travel. Only locations south of the 35° north within the previous six months were included in the analysis and were defined as travels to southern latitudes. The year was divided into two seasons; summer was defined as May-October and winter as November-April.

Dietary intake of vitamin D was calculated using four day food records. At the study visit, subjects were asked to record their food consumption for four consecutive days, including at least one weekend day, to start their record within one week of the visit and not to change their usual diet. Subjects were instructed to record the amount of all food and drinks they consumed in grams, in household volume measures or by using the portion guide “Matmallen” with photographs of portion sizes of different foods [23]. Subjects were contacted if their food records were ambiguous. Dietary intake was calculated using computer software Dietist XP version 3.1 (Kost och näringsdata, Bromma, Sweden) based on the Swedish National Food Administrations database (2009-11-17). Subjects were also asked if they consumed dietary supplements and if so the brand, dose and frequency were registered. In the statistical analysis, supplement use was defined as ≥5 µg/day and <5 µg/day as non-user. The “dietary vitamin D intake” was defined as vitamin D calculated from the food record and the “total vitamin D intake” was defined as intake from the food record plus the reported supplement use.

The subjects’ daily energy requirements were calculated using FAO/WHO/UNU’s equation for basal metabolic rate (BMR) based on body weight and age group [24]. Physical activity level (PAL) was calculated using the subjects estimation of their activity level on a scale 1–10 where 1 represented PAL 1.3 and 10 PAL 2.2 [25]. To validate the food record, food intake level (energy intake divided by BMR) was compared to the self-estimated PAL of each subject as well as PAL 1.6 (corresponding to a sedentary work and little or no strenuous leisure activity [24]) according to Goldberg et al [26] generating a range for the food intake level that is acceptable for the group. The within subject variation in BMR in the equation was set to 8.5%.

### Laboratory Analyses

Blood samples were protected from UV-B radiation and centrifuged within 45 minutes of sampling, at 5°C for 9 minutes (Centrifuge CR3i, Jouan Quality System) at 3800 g. Serum was extracted and stored at −70°C until analysis. Total 25-hydroxyvitamin D was analyzed using the vitamin D total-analysis Roche Cobas® electrochemiluminescence immunoassay. The method has a detection range of 10–175 nmol/l for 25(OH)D with a total CV of 7% for concentrations of 25 nmol/l and 4.6% for 66 nmol/l. The assay has been compared to liquid chromatography–mass spectrometry, and results show that electrochemoluminiescent immunoassay tends to give higher concentrations (y = 1.09x−0.51, r = 0.89, according to the manufacturer). Intact parathyroid hormone (PTH) was analyzed using an immunochemical two step analysis of sandwich type, using chemiluminescence microparticle immunoassay technology (Abbott laboratory diagnostics division). Total CVs were 3.7%, 4.5% and 3.5% for PTH serum concentrations of 10, 40 and 730 ng/l respectively. Analyses were performed by the Central Laboratory, Sahlgrenska University Hospital, Gothenburg, Sweden.

### Statistical Analyses

The coefficients of determination for vitamin D status were calculated using multivariate regression analysis and simple linear regression for the variables in the model: dietary vitamin D intake, supplement use, hormonal contraceptive use, BMI and variables relating to sun exposure. These included recent travel to a southern latitude, recent sunbed use, skin type, sun preference and use of sun protection factor. Height, PAL, smoking, parity and time since previous lactation, were considered potential confounders. A variable was defined as a confounder if its inclusion in the regression model caused >10% change in the regression coefficient. When comparing PTH concentrations at different cut off limits for 25(OH)D, means between groups were compared using ANOVA and post hoc Bonferroni. Student’s t-test was used to compare two means between groups. χ^2^ test was used to test for differences in proportions. A two-tailed p-value of 0.05 was considered statistically significant. All statistical analyses were performed using statistical software SPSS version 19.0 (IBM SPSS Statistics, SPSS Inc. Chicago). Mean±SD are presented unless otherwise stated.

## Results

The subjects’ characteristics are shown in table 1 and the lifestyle factors in table 2. All 84 subjects were fair-skinned women of childbearing age (33.8±3.8 years), living at latitude 57–58° north. Nineteen percent of the subjects were nulliparous. Median parity was one child (range 0–3). Seventy nine percent of the subjects had been educated to the equivalent of at least three years at university level. Four subjects smoked. Twenty seven percent had travelled to southern latitudes in the previous six months. Sixty eight percent preferred to be “both in sun and shade” and 64% claimed to “sometimes” use sun protection factor. Four of the subjects (five percent) had used a sunbed in the last 30 days. Mean dietary vitamin D intake was 5.7±3.2 µg/day and the mean total vitamin D intake 6.8±3.9 µg/day. Fifty seven percent of all blood samplings were conducted during winter.

**Table 1:** Subject characteristics of the 84 female subjects.

	Mean	95% CI	Min–Max
**Age (years)**	33.8	(32.9–34.6)	25.7–41.7
**Height (cm)**	168.4	(167.1–169.8)	153.5–183.0
**Weight (kg)**	64.3	(62.3–66.3)	48.6–94.7
**BMI (kg/m^2^)**	22.7	(22.0–23.4)	17.5–34.7
**Physical activity level**	1.8	(1.76–1.84)	1.4–2.2
**Months since partum** †	22.4	(17.8–27.0)	12–134
**Months since lactation** †	15.3	(10.5–20.1)	6–128
**25(OH)D** ‡ **(nmol/l)**	65.8	(61.4–70.1)	21.0–123.0
**PTH** § **(ng/l)**	56.7	(51.9–61.6)	20.0–160.0
**Dietary vitamin D intake (µg/day)**	5.7	(5.0–6.4)	0.3–14.1
**Supplementary vitamin D intake (µg/day)**	1.1	(0.5–1.7)	0.0–15.0
**Total vitamin D intake** ¶ **(µg/day)**	6.8	(5.9–7.6)	1.5–19.6

† Nulliparous women excluded;

‡ 25(OH)D, 25 hydroxyvitamin D;

§ PTH, parathyroid hormone;

¶ Supplementary and dietary intake of vitamin D.

**Table 2:** Lifestyle factors and determinants of vitamin D status.

Variable	N (%)	Mean 25(OH)D† nmol/l	P-value‡
**Sun protection factor use**			
Never	8 (10)	59.7	
Occasionally	54 (64)	66.1	
Always	22 (26)	67.1	0.4
**Sun preference**			
Always in sun	20 (26)	67.5	
Both sun and shade	57 (68)	65.0	
Always in shade	7 (8)	66.6	0.8
**Skin type**			
I	2 (2)	67.6	
II	15 (18)	64.7	
III	53 (63)	65.1	
IV	14 (17)	69.2	0.6
**Current estrogen contraceptive use**			
No	69 (82)	63.9	
Yes	15 (18)	74.1	0.07
**Recent travel to southern latitude** §			
No	61 (73)	62.9	
Yes	23 (27)	73.3	0.03
**Vitamin D supplement use**			
<5 µg/day	74 (88)	63.9	
≥5 µg/day	10 (12)	79.6	0.02
**Recent sunbed use** ¶			
No	80 (95)	64.9	
Yes	4 (5)	83.8	0.06
**Season**			
November–April	48 (57)	61.8	
May–October	36 (43)	71.0	0.035

† 25(OH)D, 25-hydroxyvitamin D;

‡ Significance level attained from simple linear regression;

§ Within the last 6 months;

¶ Within the last 30 days.

For all subjects, mean 25(OH)D concentration was 65.8±19.9 nmol/l. Serum 25(OH)D concentrations <75 nmol/l was found in 69% of the subjects and concentrations <50 nmol/l in 23% (table 3). Only one subject (1%) had a serum 25(OH)D concentration <25 or <30 nmol/l. Mean serum PTH was 56.7±22.3 ng/l. There was a significant negative correlation between PTH and 25(OH)D (r = −0.32, p = 0.003). Subjects with 25(OH)D concentrations <50 nmol/l had significantly higher PTH concentrations than subjects with 25(OH)D >75 nmol/l (67.4 vs. 48.4 ng/l, p = 0.014).

**Table 3:** Mean PTH concentrations at commonly used 25(OH)D cut off points for vitamin D status assessment.

25(OH)D† nmol/l	N (%)	Mean PTH‡ ng/l
**<25/30**	1 (1)	–
**<50**	19 (23)	67.5§
**50–75**	39 (46)	57.0
**>75**	26 (31)	48.4§

† 25(OH)D, 25-hydroxyvitamin D;

‡ PTH, Parathyroid hormone;

§ Significant difference in mean PTH between 25(OH)D <50 and >75 nmol/l (p = 0.014).

Simple linear regression analysis showed that significant determinants of 25(OH)D concentrations were recent travel to southern latitudes, season and use of supplementary vitamin D (≥5 µg daily) (table 4). Recent sunbed use and use of contraceptives containing estrogen were close to significance and therefore included in multivariate analysis where all variables attained significance. When examined in multivariate linear regression, the coefficient of determination for the five variables was 27%.

**Table 4:** Determinants of 25-hydroxyvitamin D concentrations: results from simple linear and multivariate regression analyses.

	Simple linear regression			Multivariate regression†		
Variables	β	SE	P	β	SE	P
**Sunbed use** ‡	18.98	10.04	0.062	21.94	9.18	0.019
**Supplement use** §	15.73	6.52	0.018	13.33	6.06	0.031
**Travel to southern latitude** ¶	10.36	4.76	0.033	10.05	4.42	0.026
**Season** ††	9.21	4.30	0.035	12.04	3.95	0.003
**Estrogen contraceptive use** ‡‡	10.26	5.59	0.070	11.66	5.06	0.024

† The multivariate model has a combined coefficient of determination of 0.27. All five variables were included in multivariate analysis.

‡ Within the last 30 days.

§ ≥5 µg/day.

¶ Within the last 6 months.

†† Summer (May–October) and winter (November–April).

‡‡ Yes/no.

Vitamin D status was significantly higher in May–October than in November–April (71.0 vs. 61.8 nmol/l, p = 0.035). Mean 25(OH)D concentration was highest in September at 77.8 nmol/l and lowest in December at 48.0 nmol/l. Only one blood sample was obtained in December whereas 3–14 observations per month were recorded during the other months. During November-April, 29% of the subjects had 25(OH)D concentrations <50 nmol/l and 79% had concentrations <75 nmol/l. Subjects who had travelled to southern latitudes had a higher mean 25(OH)D concentration than those who had not (73.3 vs. 62.9 nmol/l, p = 0.03). The subjects who had used a sunbed within the previous 30 days tended to have a higher mean 25(OH)D concentration than those who had not (83.8 vs. 64.9 nmol/l, p = 0.06). Subjects who consumed ≥5 µg/day of vitamin D supplements had a higher mean 25(OH)D concentration than subjects who did not use vitamin D supplements or had a daily vitamin D supplement intake <5 µg (79.6 vs. 63.9 nmol/l, p = 0.02). Vitamin D supplement intake ranged between 0.5–15.0 µg/day with a mean of 5.2 µg. All supplements were reported to contain cholecalciferol. Sunbeds and vitamin D supplements tended to be more commonly used during winter than summer (p = 0.08 and p = 0.07). Subjects who were currently using estrogen contraceptives tended to have a higher mean 25(OH)D concentration than non-users (74.1 vs. 63.9 nmol/l, p = 0.07). PTH concentrations were lower in estrogen users than in non-users (45.0 vs. 59.3 ng/l, p = 0.001).

Both dietary vitamin D intake and total vitamin D intake were non-significant determinants of 25(OH)D concentration. Twenty one percent of the subjects had a total vitamin D intake of ≥10 µg/day. Forty percent of the subjects had a total intake of <5 µg/day. Two of the subjects had a total vitamin D intake >15 µg/day, both were supplement users. The largest contributors of vitamin D in the food records were fish (47%), fortified margarines (15%) and fortified milk (10%). The mean reported food intake level was 1.43±0.33 for the group and the calculated range for acceptable food intake level was 1.75–1.87 using the self-estimated mean PAL of 1.8±0.2. A total of 86% of the subjects had a lower food intake level than 1.75. When instead using PAL of 1.6, 69% of the subjects reported a food intake level below the acceptable cut off limit. BMI and BMI classification were non-significant determinants of 25(OH)D concentration (p>0.05). None of the potential confounders fulfilled the criteria for inclusion in the multivariate model, nor did they attain a significant impact on 25(OH)D concentrations one-by-one.

## Discussion

This study is one of few investigating the vitamin D status in healthy, fair-skinned women of childbearing age living at northern latitudes. Data on determinants of 25(OH)D concentrations are very limited in this population.

Vitamin D status in this group of women could be considered adequate as only one subject had 25(OH)D concentration <25 nmol/l. However, 25(OH)D concentrations <50 nmol/l were found in 23% of the subjects and only 31% had concentrations >75 nmol/l. Our results are comparable to findings in women of similar age in Canada where mean 25(OH)D concentrations were 76 nmol/l during summer and 58 nmol/l during winter [21]. Data from the United Kingdom also show a slightly higher mean concentration (77 nmol/l) in women of a wider age range [27]. Previous studies on similar populations in Sweden have observed considerably higher mean 25(OH)D concentrations of 96.4 and 99.7 nmol/l [18], [19]. One can only speculate that vitamin D status among women of childbearing age may have decreased over time. Salzer et al. suggests that the proportion of pregnant women with 25(OH)D concentrations ≥75 nmol/l has declined throughout 1976–2005 in Sweden [28]. However, comparisons of 25(OH)D concentrations from different studies must be made with caution. Previous studies have used different laboratory methods for 25(OH)D assessment. This is a recognized problem in comparisons of vitamin D status between studies, populations or laboratories [29], [30], [31].

There is a negative feedback of the 25(OH)D metabolite 1,25-dihydroxyvitamin D on PTH, explaining in part why vitamin D status is important for bone turnover and bone health [32]. Accordingly, PTH and 25(OH)D concentrations were significantly correlated. In addition, mean PTH concentration was lower in the subjects with 25(OH)D concentrations >75 nmol/l compared to subjects with concentrations <50 nmol/l. Correlation between PTH and 25(OH)D is a common finding and previous studies on women of childbearing age have found similar correlation coefficients as ours [20], [21].

Significant determinants of serum 25(OH)D concentrations were recent sunbed use, season, vitamin D supplement use, estrogen contraceptive use and recent travel to southern latitudes. This multivariate model explained 27% of the variance in 25(OH)D concentrations. Only 5% of the subjects had used a sunbed during the previous month. This may explain why sunbed use only reached significance in the multivariate model. The effect of sunbed use on vitamin D status has been observed before at similar latitudes [15], [27], [33] but not in this specific population. Previous data even suggest that sunbed use three times per week might be more effective in raising 25(OH)D concentrations than vitamin D supplements of 40 µg cholecalciferol per day [34]. Vitamin D supplement use ≥5 µg/day was a significant determinant of vitamin D status. Vitamin D supplement use was quite common but many subjects did not consume the supplement daily, possibly making the intake too low to achieve an impact on 25(OH)D concentration. Previous data indicate that doses of vitamin D supplements need to be larger than 10 µg/day to maintain 25(OH)D concentrations in the absence of UV-B radiation [35], [36]. Therefore, infrequent use of supplements containing <5µg of vitamin D may have little impact on vitamin D status. Eighteen percent of the subjects used estrogen contraceptives, which was a significant determinant of vitamin D status in the multivariate model. The impact of estrogen contraceptives [37] or oral contraceptive pills [20] on 25(OH)D has been previously observed. The mechanism behind this association is theorized to be driven by the anabolic effect of estrogen on the synthesis of vitamin D binding protein, impacting the ratio of protein bound and free 25(OH)D [38]. The clinical implication of this finding is somewhat unclear, but we found that users of estrogen contraceptives had significantly lower PTH concentrations, suggesting a potential effect on bone turnover. The seasonal variation in serum 25(OH)D concentration is a common finding in previous studies [14], [20], [27], [39] and is not surprising at latitudes 57–58° north where UV-B radiation is insufficient for much of the year. Many subjects had travelled to southern latitudes during the previous six months. Those who had travelled had significantly higher mean serum 25(OH)D concentrations. The significant impact of travels to southern latitudes on vitamin D status has been observed in previous studies [14], [27], [39] but few performed in female subjects of childbearing age [20]. The seasonal variation in 25(OH)D concentration was apparent in spite of the tendency of more common use of sunbeds and vitamin D supplements during winter.

Previous studies have found that dietary intake [40], [41], BMI [20], [39], [41] and genetics [42] may influence 25(OH)D concentrations. Genetics was not investigated in this study, and we found no significant association between BMI and 25(OH)D concentrations. This may be due to the small number of obese subjects in this study. Also, no significant impact of dietary vitamin D intake on 25(OH)D concentration was observed. This has multiple possible explanations including over- or underreporting of vitamin D intake and insufficient power in dietary assessment data. It is clear that the subjects in our study were less overweight and obese, smoked less and had a higher education level than the corresponding general population [43]. The sample is therefore not representative of Swedish women in general and might illustrate a subpopulation with higher health awareness. This might in turn have influenced the mean 25(OH)D concentration since higher vitamin D status has been found to covariate with a healthy lifestyle [41]. An examination of vitamin D status and its determinants among women at northern latitudes in lower socio-economic groups is warranted.

It is interesting that three of the five significant determinants of vitamin D status were related to sun exposure. Recent publicity about the risks of skin sun damage might make people avoid sun exposure, preventing cutaneous vitamin D synthesis. Sunbed use and travels to southern latitudes are not practical recommendations to improve vitamin D status. This makes vitamin D supplement use an option for improving 25(OH)D concentrations at latitudes with recurring absence of cutaneous vitamin D synthesis. Recommendations on safe UV-B exposure could also be beneficial for vitamin D status.

### Conclusion

In conclusion, every fifth subject in this group of fair-skinned women of childbearing age at latitude 57–58° north had 25(OH)D levels <50 nmol/l. Almost 30% of the variation in 25(OH)D concentrations was explained by sun exposure, use of vitamin D supplements and use of estrogen contraceptives. Cutaneous 25(OH)D synthesis seems to be a major contributor to vitamin D status, even at northern latitudes. Thus, recommendations on safe UV-B exposure could be beneficial for vitamin D status.
